# Anharmonic and surface effects on thermodynamic properties of Ag, Al, and Cu nanostructures

**DOI:** 10.1039/d6ra06008k

**Published:** 2026-09-28

**Authors:** Hua Xuan Dat, Nguyen Thi Thao, Ho Khac Hieu

**Affiliations:** a Faculty of Physics, Hanoi National University of Education 136 Xuan Thuy, Cau Giay Ha Noi Vietnam; b Hung Vuong High School for the Gifted Viet Tri Phu Tho Vietnam; c Faculty of Physics and Chemistry, The University of Da Nang – University of Science and Education 459 Ton Duc Thang, Hoa Khanh Da Nang 550000 Vietnam hkhieu@ued.udn.vn

## Abstract

We present a theoretical approach combining the statistical moment method (SMM) and a bonding-energy model to investigate the size-, shape-, and temperature-dependent thermodynamic properties of Ag, Al, and Cu nanostructures. Analytical expressions are derived for the mean square displacement (MSD), thermal expansion coefficient, isobaric heat capacity, and Debye temperature. The results demonstrate that as the characteristic size decreases, the MSD, thermal expansion coefficient, and isobaric heat capacity increase, whereas the Debye temperature decreases. As the size increases, these thermodynamic quantities asymptotically approach their corresponding bulk values. Notably, at high temperatures, we observe pronounced nonlinear variations in nanostructures smaller than 5 nm, highlighting the critical role of anharmonic effects in lattice vibrations. Furthermore, the thermodynamic properties exhibit a strong shape dependence governed by the dimensionless shape factor that quantifies the effect of nanoparticle geometry on the surface-atom fraction. Regular tetrahedral particles show the greatest size-dependent variations due to their high shape factor, while thin films exhibit the lowest. This confirms that the surface area fundamentally drives the thermodynamic behavior of these nanomaterials. This study provides valuable theoretical insights for the design and optimization of nanostructure architectures tailored for specific practical applications.

## Abstract 2

Coupling SMM with a size- and shape-dependent bonding-energy model reveals how surface and anharmonic effects govern MSD, thermal expansion, heat capacity, and Debye temperature in Ag, Al, and Cu nanostructures.

## Graphical abstract

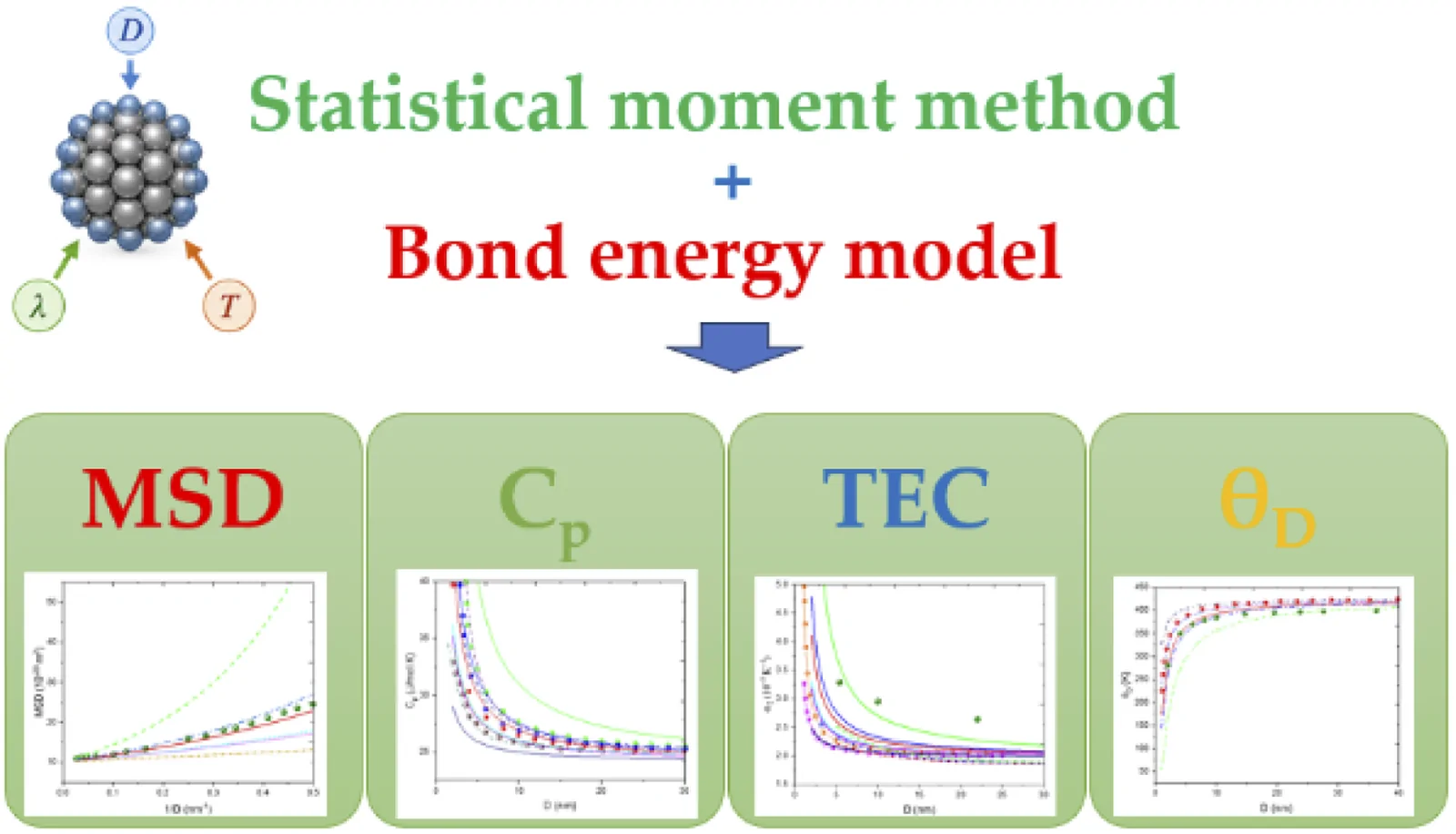

## 1 Introduction

In recent years, metal nanoparticles have become an important research focus in modern materials science due to their superior physical and chemical properties compared to bulk materials. As the material size decreases to the nanometer scale, the surface area-to-volume ratio increases sharply, leading to significant changes in electronic structure, surface energy, and atomic bonding. As a result, metal nanoparticles exhibit many outstanding properties, such as high catalytic activity, strong photoabsorption, superior electrical and thermal conductivity, and good compatibility with various application environments.^1,2^ These properties offer many opportunities for applications in chemical catalysis, biosensors, nanoelectronics, plasmonic materials, heat transfer, energy storage, and biomedicine.^3–5^ However, due to their high surface energy and lower thermal stability compared to bulk materials, metal nanoparticles also have limitations, including agglomeration, surface restructuring, and property degradation at high temperatures.^6^

Among metal nanosystems, silver (Ag), copper (Cu), and aluminum (Al) are materials of particular interest due to their wide application potential and relatively low fabrication costs. Ag nanoparticles stand out with their strong surface plasmon resonance, high antibacterial properties, and good catalytic activity, making them widely used in optical sensors, antibacterial materials, and biomedical applications.^2,3^ Cu nanoparticles have high electrical and thermal conductivity, making them suitable for nanoelectronic components, electrochemical catalysts, and efficient heat transfer materials.^1,7^ Meanwhile, Al nanoparticles have low density, high energy density, and strong reactivity, making them suitable for applications in nanofuels, energy materials, and energy storage technologies.^8^ In addition, Ag–Cu nanosystems and metal alloy nanoparticles exhibit superior properties, including structural stability, catalytic activity, and the ability to tune optoelectronic properties through nanoparticle size and shape.^5,9^ The performance and stability of metal nanosystems depend closely on thermodynamic properties such as melting temperature, Debye temperature, specific heat capacity, MSD, and thermal expansion coefficient (TEC), which are strongly influenced by size, shape, and surface effects at the nanoscale.^10–14^

Many previous studies have shown that the thermodynamic properties of metal nanoparticles depend strongly on particle size and shape.^10–24^ As particle size decreases to the nanometer range, the surface atomic ratio increases rapidly, leading to a decrease in the average bonding energy, which, in turn, results in a significant decrease in the melting temperature, Debye temperature, and consolidation energy of the nanosystem.^10,11^ Based on the modified Gibbs–Thomson model, Ansari showed that the melting temperature, Debye temperature, and specific heat capacity of metal nanoparticles vary strongly with size due to surface energy and finite-size effects.^10^ Similarly, Kareem and Mawllod also showed that many thermodynamic quantities, such as entropy, heat capacity, and melting temperature of nanoparticles, change significantly when particle size decreases below a few tens of nanometers.^12^ Besides size, the geometric shape of nanoparticles also plays a particularly important role in thermal stability and phase transition mechanisms. Recent studies have shown that spherical, cubic, polyhedral, or wire-shaped nanoparticles have different surface atomic densities and crystal face distributions, leading to significant differences in melting temperature and lattice vibration properties.^16–18^ Delgado-Callico *et al.* discovered a “universal sign” in the melting of metal nanoparticles, showing that the melting mechanism strongly depends on the shape and surface structure of the nanoparticles.^17^ Meanwhile, Ansari developed theoretical models describing the simultaneous dependence of Debye temperature, melting entropy, and melting enthalpy on the size and shape of metal nanomaterials.^19^ Recent studies on Ag–Cu nanoclusters also indicate that changes in shape and composition can significantly alter the structural, vibrational, and thermodynamic properties of the nanosystem.^20^

Besides the melting temperature and Debye temperature, other thermodynamic quantities, such as MSD, isobaric heat capacity, and TEC, have also received attention recently. Thermodynamic vibration models show that the specific heat capacity and TEC of nanomaterials generally increase with decreasing size due to increased thermal vibration of surface atoms.^21,22^ In particular, Tam *et al.* showed that the MSD of nanoparticles increases sharply with decreasing size or with increasing temperature, reflecting increased atomic mobility and decreased structural stability at the nanoscale.^13,14^ In addition, Rigaut and Calvo also demonstrated that the Debye temperature and “breathing mode” vibration of metal nanoparticles strongly depend on the shape and composition of the nanosystem.^15^

Besides theoretical and simulation studies, many experimental works^25–27^ have also confirmed the strong dependence of thermodynamic properties on the size and structure of metal nanoparticles. One of the earliest and most important experimental studies was conducted by Rupp and Birringer,^25^ in which the specific heat capacity of crystalline nanomaterials was measured over the temperature range 150–300 K. The results showed that the specific heat capacity of the nanosystem increased significantly compared to that of bulk materials, due to the increased density of vibrational states in the surface region and the influence of low-energy vibrational modes. Subsequently, Qian *et al.*^26^ studied the influence of crystal lattice microstrains on the thermal properties of Cu nanocrystals. Experimental results showed that the presence of microstrains significantly altered the nanosystem's heat capacity and thermal stability by altering the lattice structure and atomic bonding energy. More recently, Bogatyrenko and Kryshtal^27^ used *in situ* TEM diffraction coupled with electron energy loss spectroscopy (EELS) to determine the TEC of Ag and Cu nanoparticles. The results showed that the TEC of the nanoparticles differed significantly from that of the bulk material and depended strongly on particle size and surface state.

Despite significant advances, most current research still focuses primarily on investigating individual thermodynamic parameters or considering only the influence of size or shape on a specific nanosystem. Some theoretical models are simple and easy to apply, but do not fully capture the simultaneous effects of size, shape, and temperature on nanoparticles' thermodynamic properties. Meanwhile, molecular dynamics simulations, although highly accurate, often require high computational costs and are heavily dependent on the inter-atomic interaction potential. Therefore, developing a theoretical model capable of simultaneously investigating the influence of size, shape, and temperature on the thermodynamic properties of Ag, Cu, and Al nanoparticles remains a scientifically and practically significant problem. In this work, we propose a theoretical model that allows the simultaneous determination of the effects of temperature, shape, and size on the thermodynamic properties of nanoparticles. The main idea is that we develop a new theoretical approach by combining the SMM^28–31^ with a size- and shape-dependent bonding energy model^32^ to build a unified computational framework for nanoparticles. In this framework, the simultaneous influence of temperature, size, and shape is incorporated by varying the atomic coordination environment, bonding energy, and lattice vibrational characteristics. On this basis, important thermodynamic properties, including MSD, TEC, isobaric heat capacity, and Debye temperature, are systematically investigated on Ag, Al, and Cu nanoparticles of different sizes and shapes.

## 2 Theoretical model

### 2.1 SMM method

Let us consider a system having *N* atoms, and the interaction between atoms is *ϕ*(*r*). With the result of thermal lattice vibrations, the zeroth central atom is affected by an additional force *f*_*β*_. And, we obtain the following force balance equation^28^1
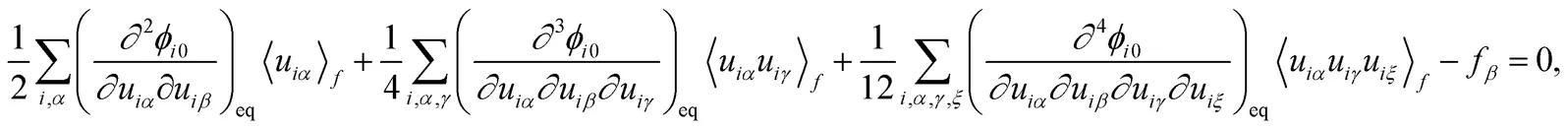
where *α* ≠ *β* ≠ *γ* ≠ *ξ* = *x*, *y*, *z* in the Cartesian coordinate system, 〈*u*_*iα*_〉, 〈*u*_*iα*_*u*_*iγ*_〉, 〈*u*_*iα*_*u*_*iγ*_*u*_*iξ*_〉 are, correspondingly, the first, second, and third order moments of the atomic displacement. A key advantage of the SMM is that it establishes a direct relationship between second-order and third-order moments, and first-order moments through the following formulas^28^2
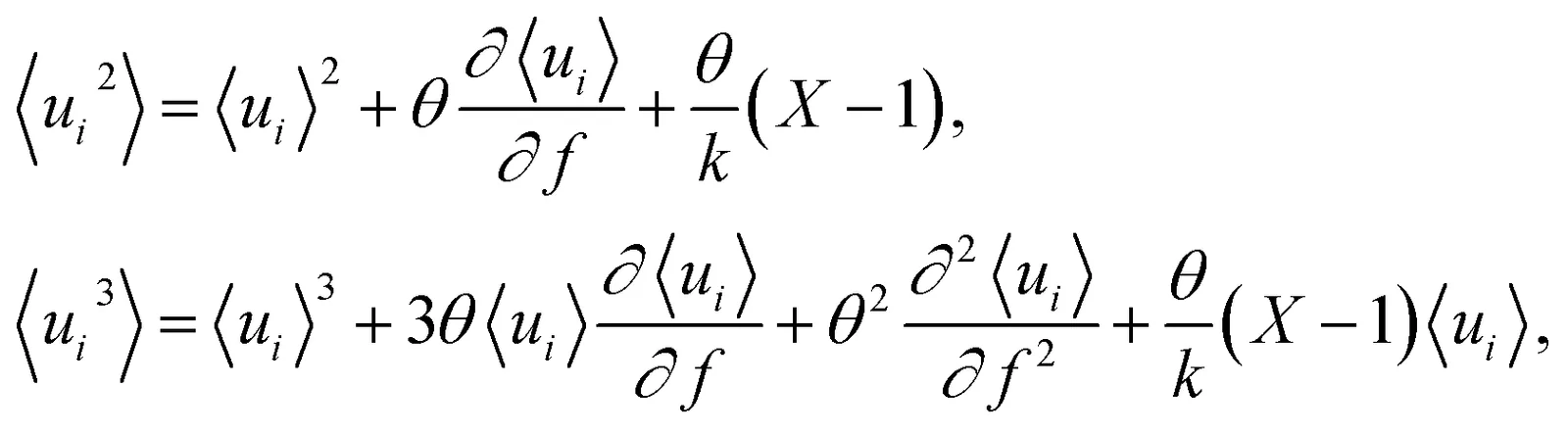
where 
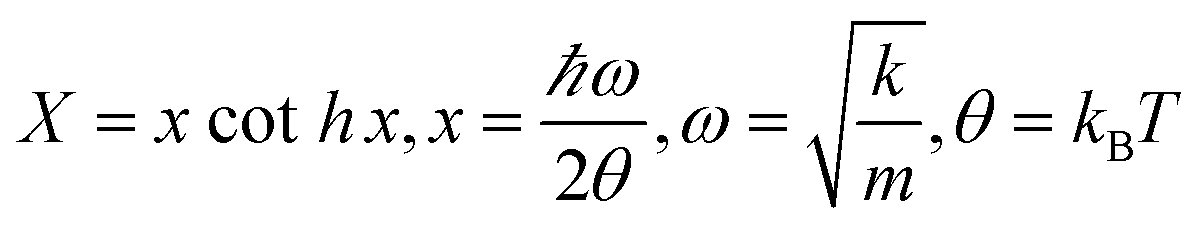
. By combining eqn (1) and (2) and setting *δ* = 〈*u*_*i*_〉_*f*_, we obtain the force balance equation in the following simple form^28,31^3
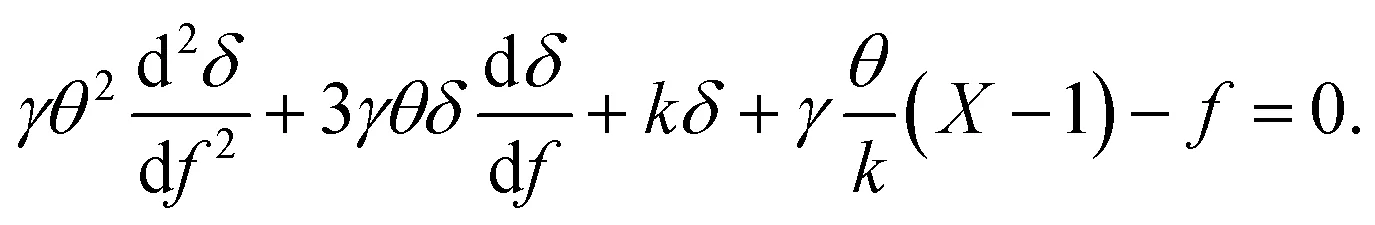
Here, *k* represents the effective harmonic force constant associated with the local vibrational stiffness, and anharmonic parameters *γ*_1,_*γ*_2_ in the form as^28–31^4
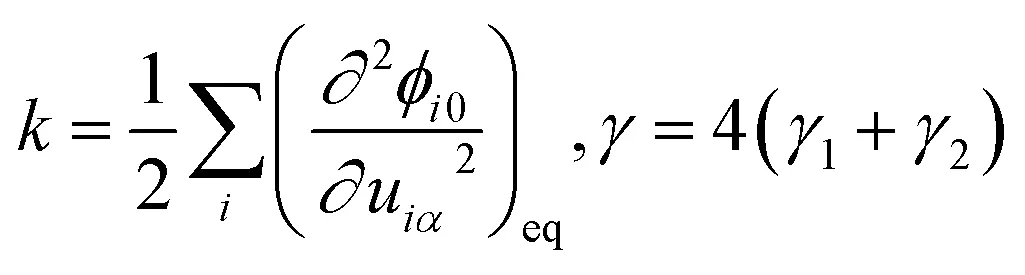
5
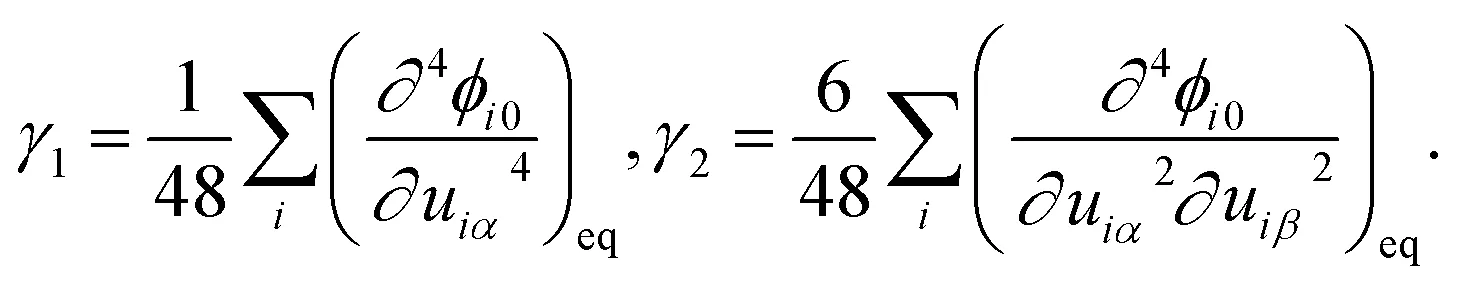


Since the external force *f* is very small, combined with the iterative solution method, we find the solution of the approximate differential equation as^28^

6 *δ*(*T*) ≈ 〈*u*_*i*_〉 + *B*_1_*f* + *B*_2_*f*^2^

with7
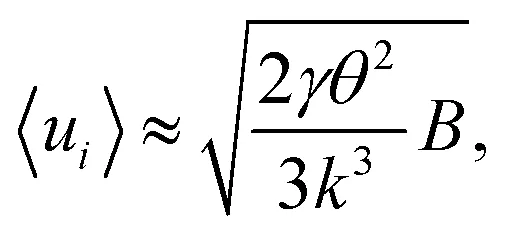
where8
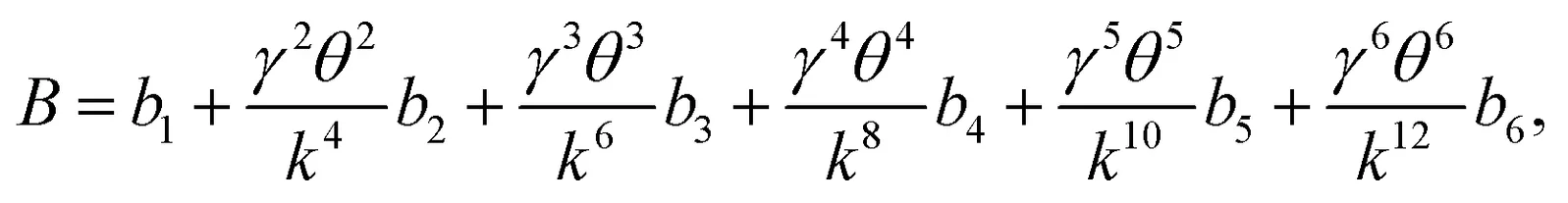
and *b*_*i*_(*i* = 1,…,6) has the form as in ref. 28. Substituting eqn (6) into eqn (2), we obtain the MSD of the crystal, which has the form as9
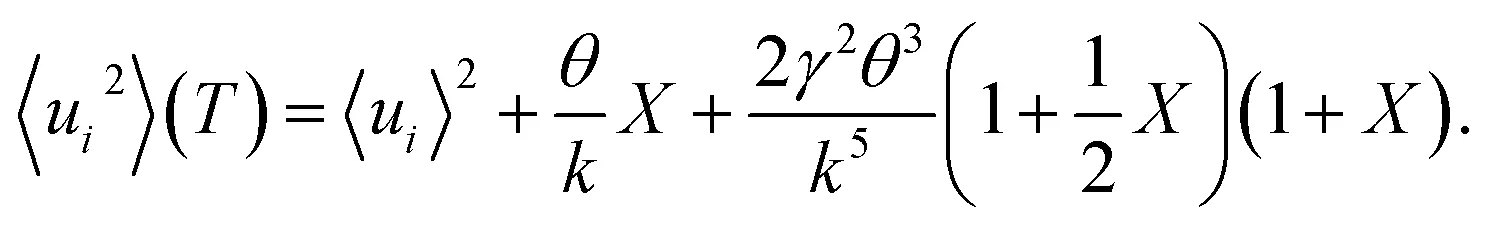


In SMM theory, the mean interaction potential of FCC crystals is determined by ref. 28 and 3110
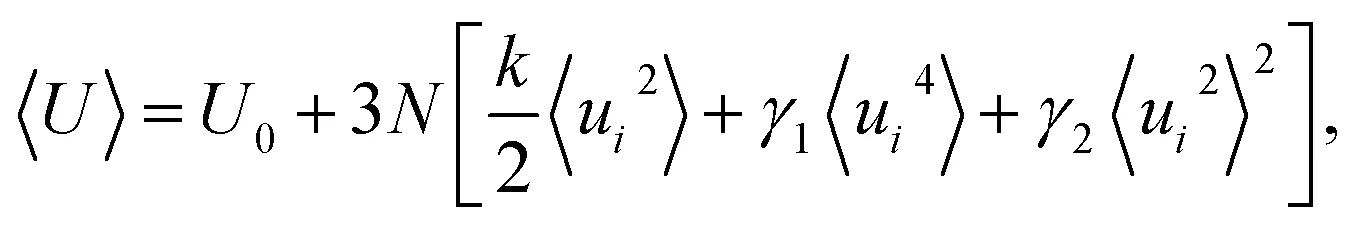
where 
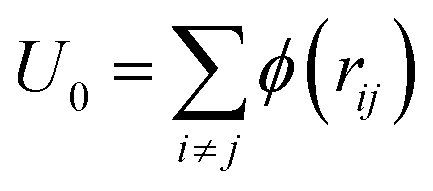
. To determine the free energy from eqn (10), we need to perform the following integral calculations11
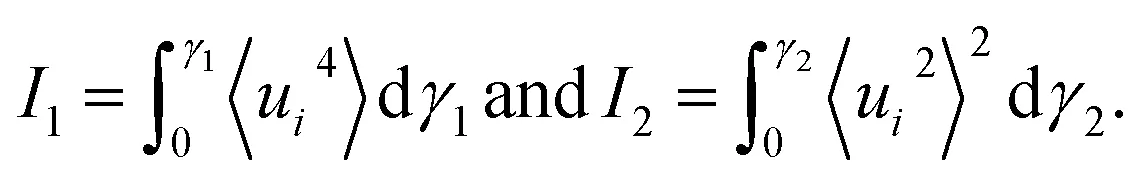


Substituting the second and fourth moment expressions^29^ into eqn (11), we obtain the expression for the free energy of the crystal system in the form12
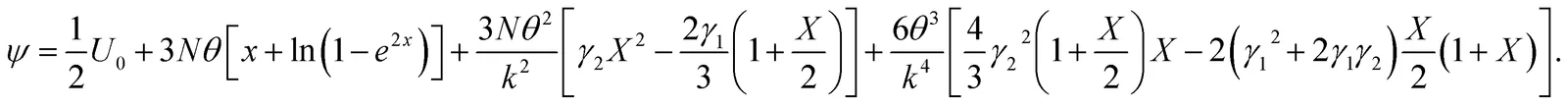


Once the free energy of the system is known, we can determine the expressions for the isothermal compression coefficient, TEC, isochoric heat capacity, and isobaric heat capacity as^31^13
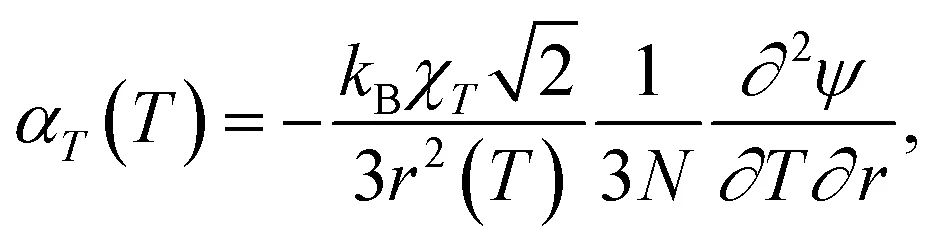
14
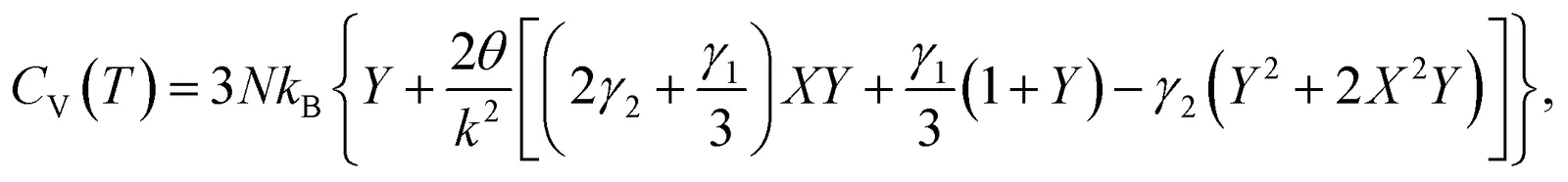
15
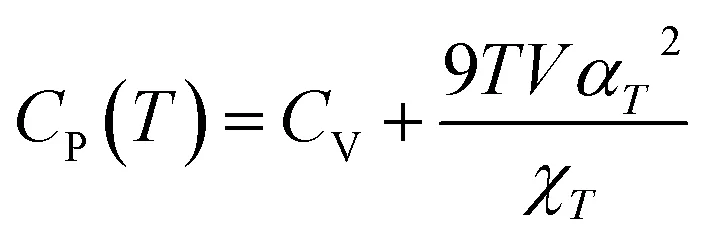
with 
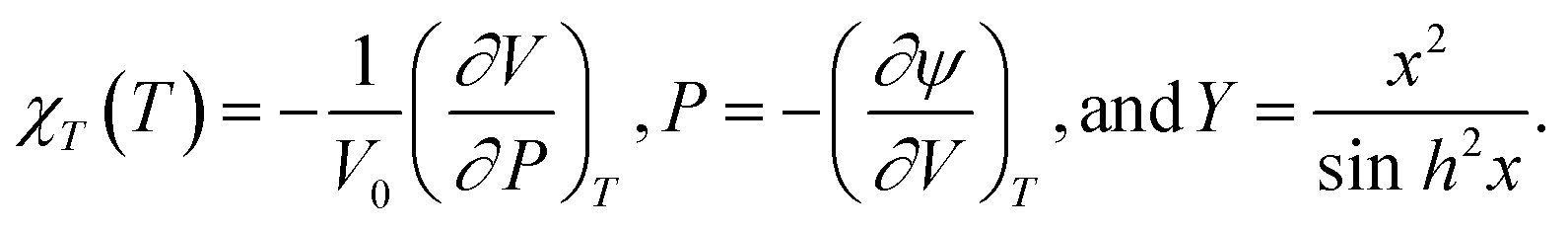


Within the SMM framework, the Einstein frequency provides a characteristic effective vibrational frequency. The Debye temperature is then obtained through the established Einstein–Debye frequency relation adopted for monatomic FCC crystals.^33^ This relation is an effective mapping between the characteristic Einstein frequency and the Debye frequency rather than an assumption that the Einstein and Debye frequencies are identical. For the FCC crystal structure, the Debye temperature is given by ref. 3316
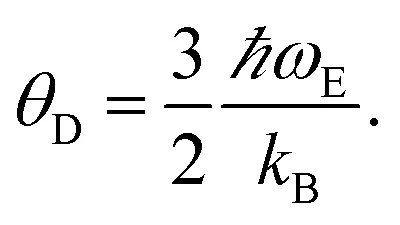


Thus, by applying SMM to Ag, Al, and Cu crystals, we can completely determine thermodynamic properties such as MSD, TEC, isobaric heat capacity, and Debye temperature for bulk materials at zero pressure and temperature *T*. From the results obtained above, we further develop the SMM theory to determine the thermodynamic properties of nanoparticles, as presented in the following section.

### 2.2 Bond energy model

According to the Lindemann melting criterion, the melting temperature is proportional to the effective force constant of the lattice vibration where the former can be expressed by the cohesive energy^34^

17 *T*_m0_ = *αE*_0_

with *α* = *nf*^2^/(3*k*_B_*Z*), where *n* is the exponent of the repulsive part of the interaction potential between constituent atoms, *f* is the ratio of the atomic displacement at *T*_m0_ to the interatomic separation at equilibrium, *k*_B_ is the Boltzmann constant, and *Z* is the valency of the atoms. When the nanocrystals have the same structure of the corresponding bulk,^35^*α* can be assumed to be size-independent as a first-order approximation. In this case, Lu *et al.*^32^ extended eqn (17) to the nanometer size with the same form. From there, the basic relationship between melting temperature and bond energy takes the form^32^18
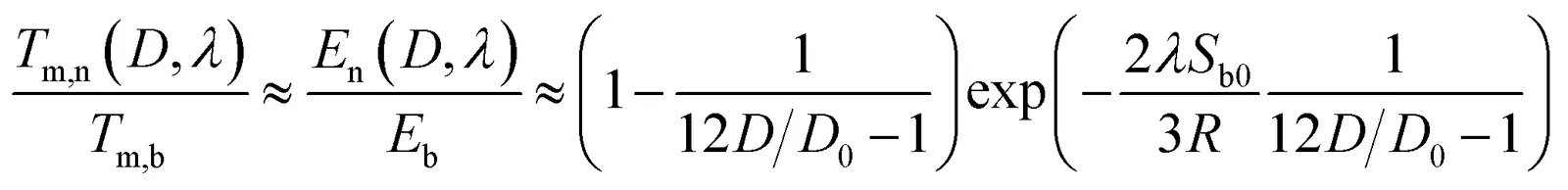
where the subscripts “n” and “b” denote a nanomaterial and bulk material, respectively. *D* is the particle size, and *d* is the dimension of the nanocrystal. *D*_0_ is the maximum size to become bulk material and has a relationship between *h*, the atomic diameter, and *D*_0_ = 2(3 − *d*)*h*. *S*_b0_ = Δ*H*_b0_/*T*_b0_ is the liquid–gas phase transition entropy, Δ*H*_b0_ is the liquid–gas phase transition enthalpy, and *T*_b0_ is the evaporation temperature. *λ* is a dimensionless shape factor that quantifies the effect of nanoparticle geometry on the surface-atom fraction.

Based on Lindemann's criterion, Shi derived a relationship between the melting temperature and MSD in nanocrystals^36^ as follows19
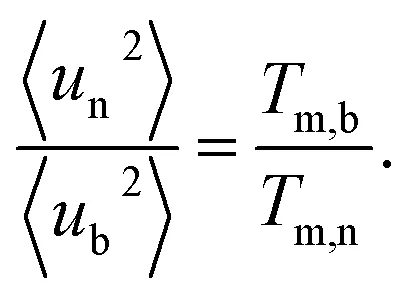


Combining eqn (9), (18) and (19) we obtain the expression to determine MSD depending on shape, size, and temperature as follows20
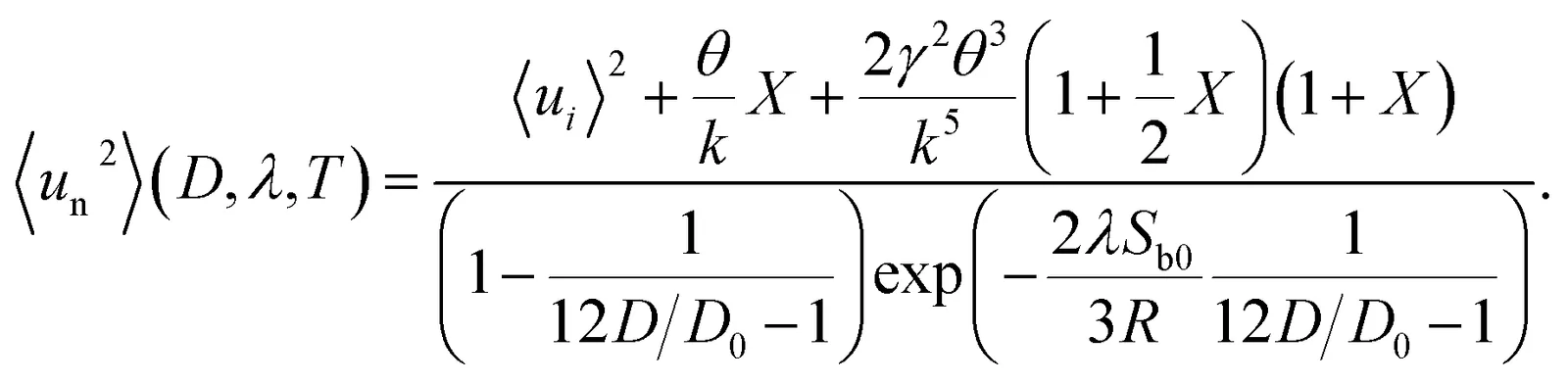


Next, Zhu *et al.*^37^ demonstrated that the TEC depends on the size and shape of the nanomaterial as21
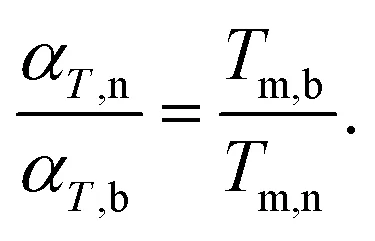


Therefore, combining eqn (9), (18) and (21) we get the TEC of the nanomaterial as follows:22
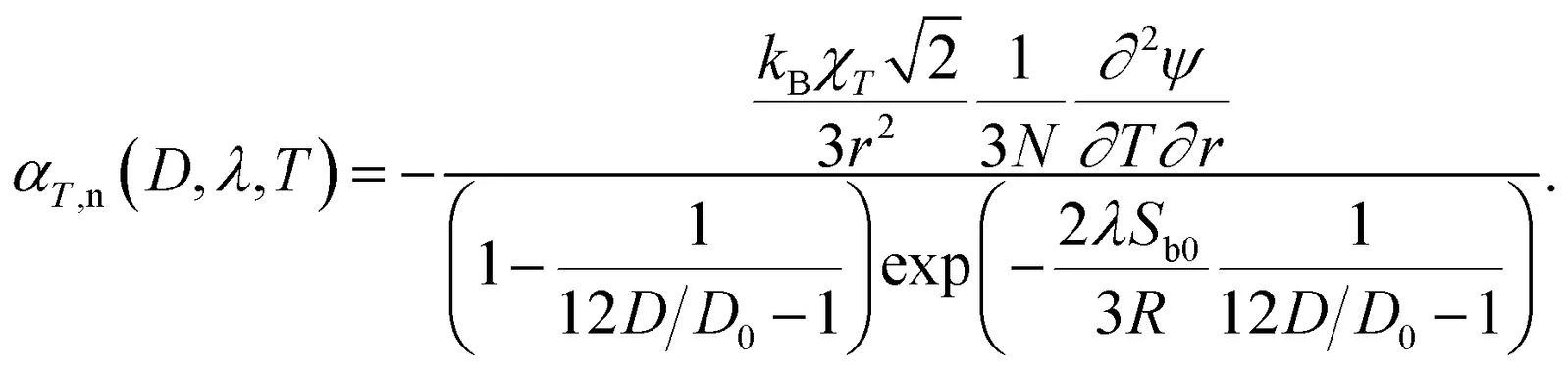


During the study of the melting behavior of bulk materials, Lindemann established a relationship between the melting point *T*_m,b_ and the Debye temperature *θ*_D,b_, expressed a
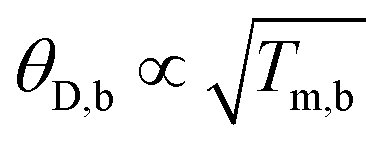
s.^38,39^ If the same relationship between the melting temperature and the Debye temperature remains true at the nanoscale 
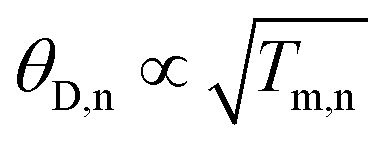
, combined with eqn (16) and (18), we derive the expression for the Debye temperature *θ*_D,n_ of nanoparticles as follows23
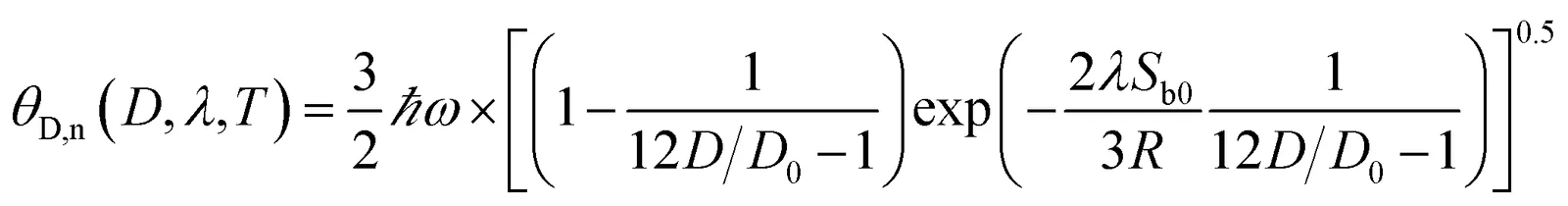


Based on Debye theory, the relationship established between the specific heat capacity at constant pressure and the Debye temperature of bulk material^40^ is 
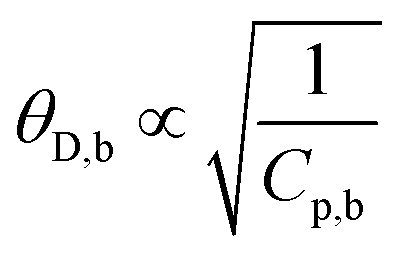
. Combining eqn (15) and (23), we get the isobaric heat capacity expression for nanoparticles as follows24
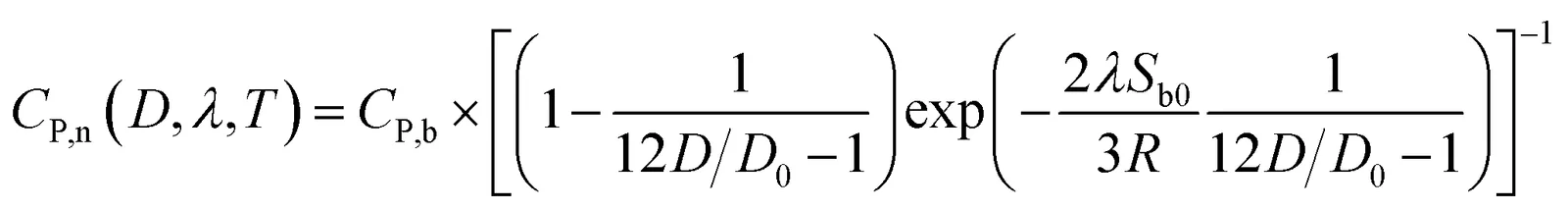


Thus, by using eqn (20) and (22)–(24), we can simultaneously determine the influence of temperature, size and shape on the thermodynamic properties of Ag, Al and Cu nanoparticles. In the next part, we will calculate the specific numbers and compare the calculated results with experimental results along with some other thermodynamic models.

It should be noted that the nanoparticle morphologies considered in this work are idealized and prescribed geometries. In real metallic nanoparticles, particularly for tetrahedral and octahedral structures, the morphology may be metastable and can evolve with temperature as surface atomic diffusion becomes activated. Experimental observations on Pt nanoparticles have demonstrated that tetrahedral and octahedral structures can undergo progressive surface reconstruction upon heating and eventually transform toward more stable equilibrium-like morphologies at temperatures below the bulk melting point.^41^ Therefore, the present calculations do not describe temperature-induced shape reconstruction. Instead, they describe the temperature dependence of the thermodynamic properties for a given fixed morphology characterized by its shape factor *λ*. This approximation is appropriate for investigating the intrinsic shape effect within the analytical model, but it should be kept in mind when interpreting the calculated results at elevated temperatures. The present model is not intended to predict the size-dependent equilibrium morphology of metallic nanoparticles. In particular, Marks decahedral and FCC truncated-octahedral motifs, which may compete with icosahedral structures depending on particle size, are not explicitly considered in the present set of prescribed geometries.

## 3 Numerical results and discussion

In the FCC structure of Ag, Al, and Cu metals, the main contribution is the pair interaction potential. In this work, to describe the bonding energy between atoms, the Morse pair interaction potential *φ*_*ij*_ = *D*{exp[−2*α*(*r*_*ij*_ − *r*_0_)] − 2 exp[−*α*(*r*_*ij*_ − *r*_0_)]} has been chosen. The Morse potential was selected because it effectively describes anharmonic interatomic interactions and has been widely used for noble metals.^42^ The Morse potential parameters for Ag, Al, Cu, and the liquid–gas phase transition entropy values *S*_b0_, and atomic diameter *h* are shown in Table 1. The shape coefficients *λ* for spheres, regular tetrahedra, regular octahedra, regular icosahedra, cylindrical nanowires and thin films are determined in ref. 32.

**Table 1:** Morse potential parameters and *S*_b0_, *h* values for Ag, Al, Cu^43,44^

	*D* (eV)	*α* (Å^−1^)	*r*_0_ (Å^−1^)	*S*_b0_ (J mol^−1^ K^−1^)	*h* (nm)
Ag	0.3323	1.369	3.115	104.72	0.289
Al	0.33409	1.39979	3.09349	104.94	0.3165
Cu	0.35279	1.40717	2.82542	93.75	0.255

First, we calculated the MSD of Ag, Al and Cu nanoparticles according to eqn (20). Fig. 1a–c respectively show the dependence of MSD on particle size for Al, Ag and Cu nanoparticles with different shapes such as spherical, tetrahedral, octahedral, icosahedral, cylindrical nanowire and nanofilm. The calculated results were compared with the theoretical data of Sadaiyandi.^45^ It can be seen that for all three materials, MSD increases rapidly as particle size decreases to a few nanometers and approaches the value of the bulk material as particle size increases. For example, the MSD of spherical Al nanoparticles increases from about 0.0118 Å^2^ to 0.0229 Å^2^ as the particle size decreases from about 10 nm to 2 nm. Meanwhile, for spherical Ag nanoparticles, MSD increased from 0.0108 Å^2^ to 0.0196 Å^2^. The results also showed that MSD depends strongly on nanoparticle geometry. Nanowire and nanofilm structures have lower MSD values than polyhedral structures such as tetrahedra or regular icosahedra. This suggests that the thermal vibration of the nanosystem depends not only on the surface area-to-volume ratio but also on the degree of atomic arrangement and the distribution of coordination numbers within each geometric structure. In polyhedral structures, the presence of many edges and vertices increases the density of low-coordination atoms, leading to a decrease in local bonding energy and an increase in atomic thermal vibration. Physically, the increase in MSD at small sizes stems from the characteristic surface effect of the nanosystem.^13,14,45^ As particle size decreases, the surface atomic ratio increases rapidly, reducing the average atomic coordination number and softening lattice vibrations, thereby increasing the atom's thermal vibration amplitude. Comparison with the results of Sadaiyandi^45^ shows that the current model fits well in terms of the variation trend and the quantitative value of MSD, especially in the medium- and large-sized regions. Small differences appear in the very small region, which may be related to differences in the bonding-energy model and the way the shape factor is defined. The research results show that MSD is closely related to the thermal stability, atomic diffusion capacity, and melting temperature of metal nanoparticles. This has important implications for the applications of Ag, Cu and Al nanoparticles in catalysis, sensors and nanoelectronic components.

**Fig. 1:**
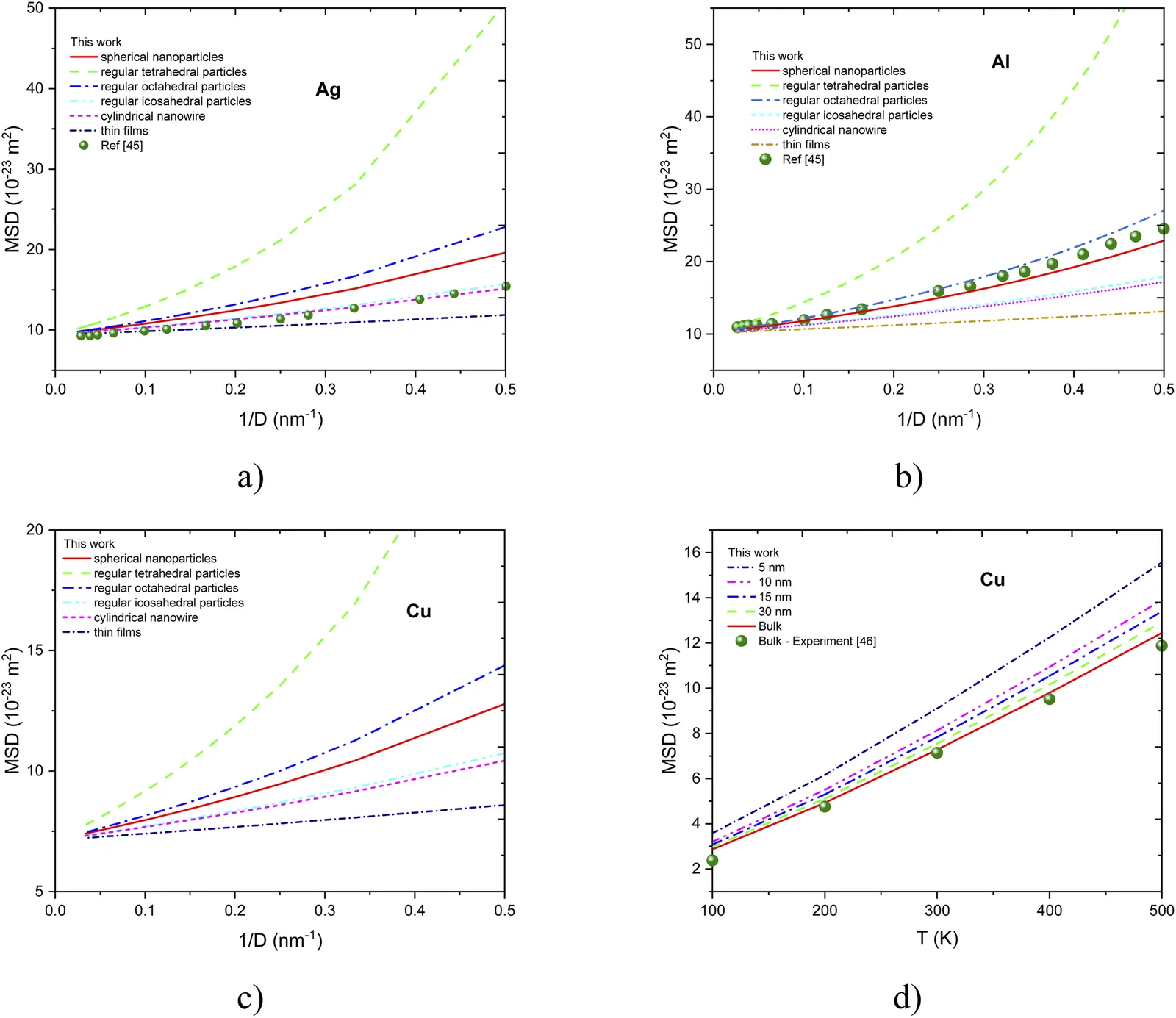
MSD of Ag, Al, Cu nanoparticles depending on shape and size at temperature *T* = 300 K are shown in figures (a–c) respectively, compared with theoretical calculations.^45^ Figure (d) shows the dependence of MSD on temperature – size of spherical copper nanoparticles and compares with experimental results.^46^

Fig. 1d presents the temperature dependence of the MSD for metal nanoparticles of sizes 5–30 nm and compares it with experimental data for bulk materials.^46^ It can be seen that, for all sizes investigated, MSD increases with temperature over the range 100 K to 500 K, and the trend becomes distinctly nonlinear at higher temperatures. This increase reflects the increase in the thermal vibrations of the atom as the system's thermal energy increases. In particular, for 5 nm nanoparticles, the MSD curve shows a significantly stronger nonlinear increase than for larger sizes at temperatures above 300 K. This indicates that the anharmonic effect of lattice vibrations becomes important at small nanoscale sizes and cannot be ignored. Comparison with experimental data^46^ shows that the current model fits quite well across the entire temperature range investigated. This confirms that the theoretical model is reliable in predicting subsequent thermodynamic properties.

Fig. 2a–c present the dependence of TEC on size for Ag, Al, and Cu nanoparticles of different shapes such as spheres, tetrahedra, octahedra, icosahedra, cylindrical nanowires, and nanofilms at 300 K. The calculated results in this study are compared with previous theoretical models^47–50^ and experimental data.^26,27,51^ It can be seen that, for all three materials, TEC increases sharply as particle size decreases to a few nanometers and gradually approaches the value of the bulk material as particle size increases. This trend is particularly pronounced in the smaller size range of about 10 nm, where the slope of the TEC curve increases rapidly as the size effect becomes strongly dominant in the nanosystem's thermodynamic properties. For example, for Al, the TEC of tetrahedral particles at approximately 2–3 nm is many times larger than that of bulk materials, while in the larger 20–30 nm region, the curves begin to converge towards values close to those of bulk materials. The results also show that TEC depends significantly on nanoparticle shape. For all three materials, tetrahedral structures generally yield the largest TEC values, followed by octahedral and icosahedral structures, while nanowires and nanofilms yield significantly lower TEC values. This reflects differences in the structural stability and the distribution of atomic coordination numbers in each geometry. Polyhedral structures contain many atoms at edges and vertices with low coordination numbers, which degrade local bonding energy and enhance atomic thermal vibrations, thereby increasing the coefficient of thermal expansion. Conversely, in nanowires and nanofilms, the arrangement of atoms along crystal planes is more stable, limiting thermal vibrations and reducing the system's thermal expansion. Physically, the increase in TEC at small sizes mainly stems from the surface effects characteristic of nanomaterials. As particle size decreases, the proportion of surface atoms increases rapidly, reducing the average atomic coordination number and weakening the effective atomic bonds. This leads to a softening of lattice vibrations and an increase in the amplitude of atomic thermal vibrations, thereby increasing the thermal expansion of the nanosystem. For very small particles, atoms at edges, vertices, and surfaces vibrate significantly more strongly than atoms in the crystal core due to their unsaturated bonding environment and lower coordination number. In addition, Al exhibits a larger TEC than Ag and Cu under the same size conditions because it has weaker atomic bonds and a lower Debye temperature, leading to stronger atomic thermal vibrations. Comparison with experimental results^26,27,51^ shows that the current model reproduces the trend in TEC variation with nanoparticle size and shape for Cu nanoparticles, especially in the medium- and large-size regions. However, significant differences remain in the very small size range below about 5–10 nm, especially for Ag nanoparticles across the entire investigated region. The results in Fig. 2 were investigated at 300 K, so the influence of anharmonic oscillations does not dominantly govern the nanosystem. Therefore, the difference between theory and experiment in this case mainly stems from structural factors and model limitations, rather than from strong anharmonic effects. First of all, most theoretical models^47–50^ assume that nanoparticles have an ideal crystal structure, a perfect geometric shape and uniform atomic distribution. These factors can significantly alter the local atomic coordination number and bond stiffness at the nanosurface. In addition, the difference between theory and experiment may also be related to experimental conditions such as lattice defects, deformation,^26^ surface state, or interactions between nanoparticles and the surrounding environment.^27^ Meanwhile, the current theoretical model assumes that nanoparticles are free, have an ideal crystal structure, and does not fully consider these external influences. This is an important reason for the difference between theoretical and experimental results in the very small region.

**Fig. 2:**
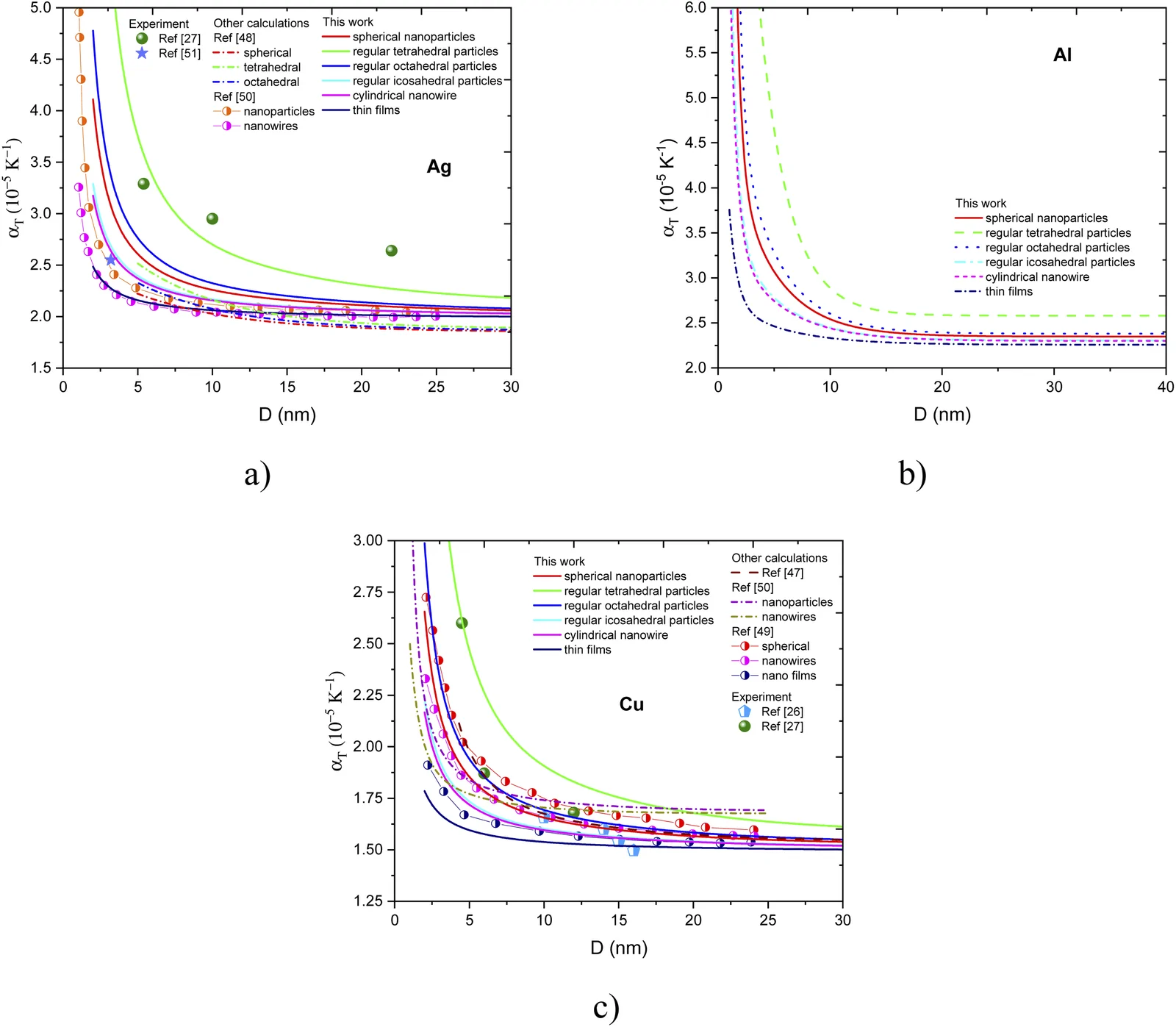
The TEC of Ag, Al, and Cu nanoparticles depends on their shape and size at temperature *T* = 300 K, respectively, as shown in figures (a–c), compared with theoretical calculations^47–50^ and experiments.^26,27,51^

Fig. 3a investigates the effect of shape on the TEC of 3.2 nm Ag nanoparticles and compares it with the experiment of Dubiel *et al.*^51^Fig. 3b describes the dependence of TEC on temperature and size for spherical Al nanoparticles compared with the experiment of Wang and Reeber.^52^Fig. 3c presents the TEC of 12 nm Cu nanoparticles with different shapes and compares it with the theoretical model of Goyal and Gupta.^49^ The results show that for all three materials, TEC increases with temperature throughout the entire investigated domain. However, this increase is nonlinear, especially at high temperatures, reflecting the influence of anharmonicity in lattice vibrations. For Ag and Cu, regular tetrahedral structures yield the largest TEC values, followed by regular octahedral, spherical, and regular icosahedral structures, whereas nanowires and nanofilms exhibit significantly lower TEC values. For example, for Cu nanoparticles at 1000 K, the TEC of the tetrahedral structure reaches nearly 2.1 × 10^−5^ K^−1^, while the nanofilm is only 1.8 × 10^−5^ K^−1^. A similar trend is observed for Ag at 3.2 nm. For the spherical Al nanoparticles in Fig. 3b, our calculations demonstrate that the TEC increases with increasing temperature and simultaneously increases sharply with decreasing size. For example, at around 600 K, the TEC of the 5 nm particle is significantly larger than that of the 30 nm particle and the bulk material. This trend clearly reflects the influence of the size effect on the thermal vibration of atoms and lattice softening at the nanoscale. Compared to Ag and Cu, Al exhibits a larger TEC due to weaker atomic bonding and a lower Debye temperature, leading to stronger atomic thermal vibration at the same temperature. Comparison with experimental data^51,52^ and the theoretical model^49^ shows that the current model yields consistent results, both in terms of the trend in TEC and its quantitative values. This has important implications for the design of high-temperature nano-components where thermal stress and structural stability directly affect material performance and lifespan. These results also provide a theoretical basis for optimizing Ag, Al, and Cu nanomaterials for microelectronics, nanothermal transfer, and thermal sensing applications.

**Fig. 3:**
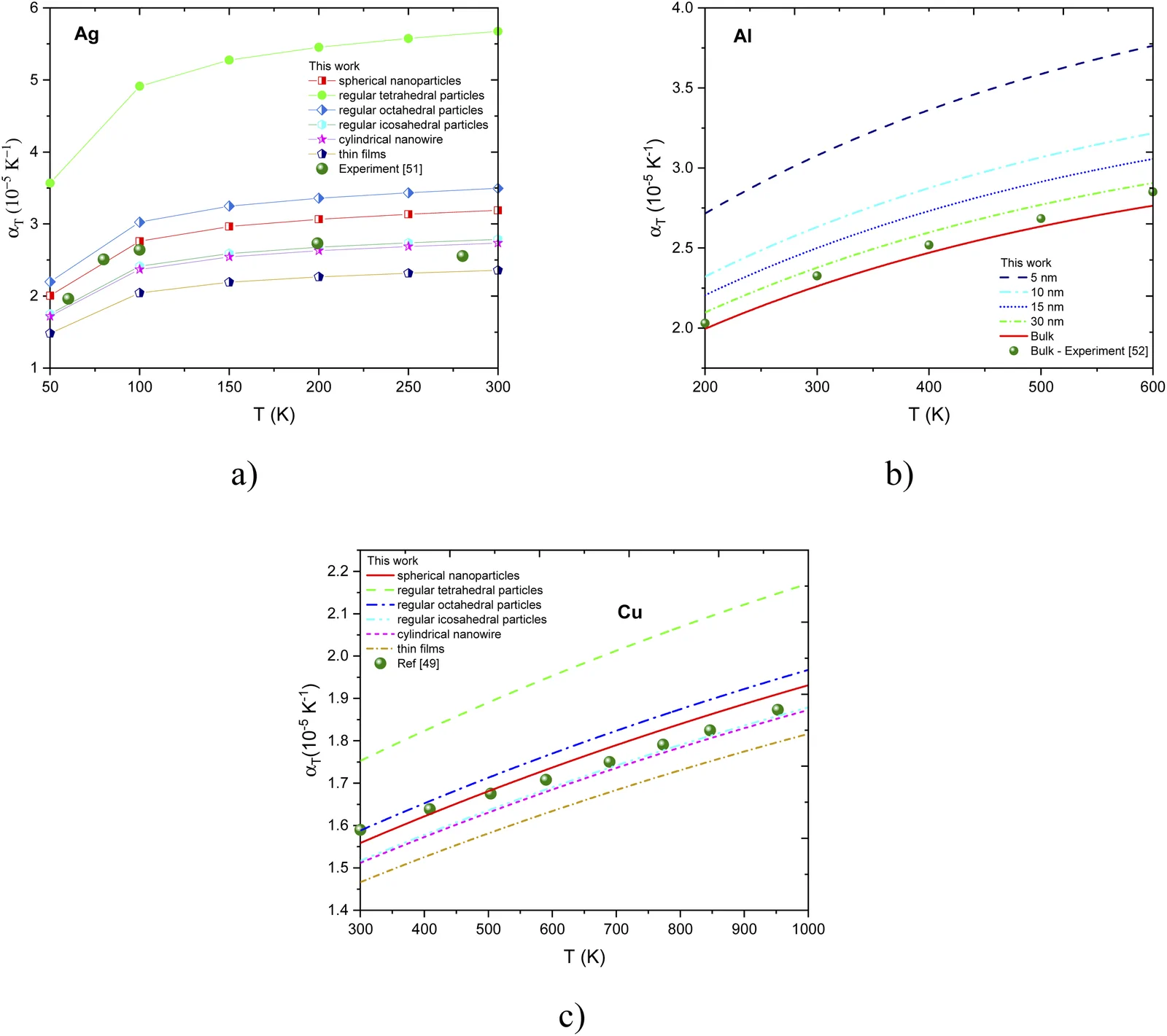
(a) TEC of Ag nanoparticles depending on shape and temperature at 3.2 nm size compared with experimental,^51^ (b) TEC of spherical Al nanoparticles depending on size and temperature compared with experimental,^52^ (c) TEC of Cu nanoparticles depending on shape and temperature at 12 nm size compared with theoretical calculation.^49^

Fig. 4 presents the Debye temperature dependence on size for Ag, Al, and Cu nanoparticles of different shapes at *T* = 300 K, including spherical, tetrahedral, octahedral, icosahedral, cylindrical nanowires, and nanofilms. The calculated results are compared with previous theoretical models.^45,53–56^ It can be seen that, for all three materials, the Debye temperature decreases sharply as the particle size decreases to a few nanometers and gradually converges to bulk material values as the size increases. This trend becomes particularly pronounced in the size range below approximately 10 nm, where the slope of the curves changes significantly due to the dominant influence of surface effects on lattice vibrations and atomic thermal dynamics. A noteworthy feature is that the Debye temperature depends strongly on the nanoparticles' geometry. The theoretical curves indicate that nanofilms and nanowires consistently exhibit the highest Debye temperatures, followed by octahedral and tetrahedral structures, while spherical and icosahedral structures exhibit lower Debye temperatures. For Cu and Al, this difference is particularly pronounced in the sub-5 nm size region, where the Debye temperature of nanofilms is significantly higher than that of spherical structures. This reflects the inherent difference in lattice vibrational stiffness between nanostructures with different anisotropies. Physically, the Debye temperature is directly related to the effective lattice stiffness and the system's characteristic vibrational frequency. As particle size decreases, the average atomic coordination number decreases due to a sharp increase in the surface atom ratio, reducing the effective bonding energy between atoms. This leads to lattice vibrational softening and a decrease in the characteristic vibrational frequency, thereby reducing the nanoparticle's Debye temperature. However, the degree of this decrease depends strongly on the nanoparticle's shape.

**Fig. 4:**
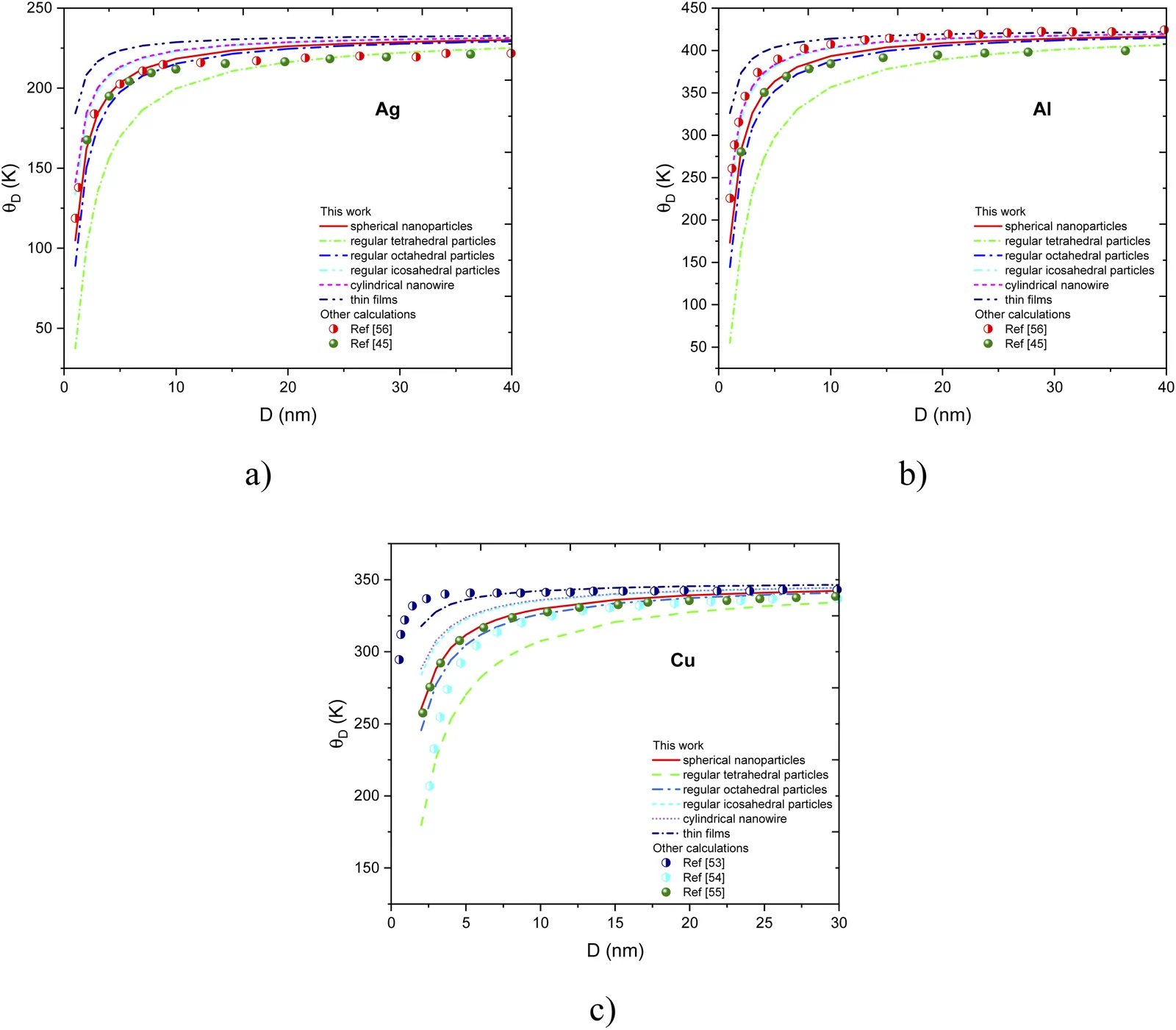
Debye temperatures of Ag, Al, Cu nanoparticles depending on shape and size at temperature *T* = 300 K are presented in figures (a–c) respectively compared with theoretical calculations.^45,53–56^

For anisotropic structures such as nanofilms and nanowires, atoms maintain a periodic arrangement along preferred crystal orientations, thereby preserving atomic bond stiffness along certain vibrational directions. Therefore, these structures exhibit considerably higher Debye temperatures than near-spherical or polyhedral nanoparticles, which contain a larger fraction of atoms at edges and vertices. In contrast, regular icosahedral and spherical nanoparticles possess a high density of low-coordinated surface atoms, particularly at edges, vertices, and highly curved surface regions, whose proportion increases markedly with decreasing particle size. These atoms experience unsaturated bonding environments and reduced coordination numbers, resulting in enhanced thermal vibrations and local lattice softening. Consequently, the effective bond stiffness is weakened, leading to a reduction in the Debye temperature. The present model accounts for the dominant average effect of this non-bulk coordination through the shape-dependent effective bonding energy, but does not explicitly resolve the distinct FCC/HCP/twin local environments or the associated fivefold strain energy. This reduces the Debye temperature of the nanosystem compared to structures with higher symmetry and stronger bonds. Comparison with previous theoretical results ^45,53–56^ shows that the current model reproduces both the variation trend and the quantitative value of the Debye temperature quite well. The results are in particularly good agreement with the models of Xiong *et al.*^56^ and Zhang *et al.*^53^ in the medium- and large-size regions. However, in the very small region below about 3 nm, certain discrepancies still exist between the models. Therefore, more advanced experimental techniques will be needed in the future to verify the above results.

Fig. 5 presents the dependence of isobaric heat capacity *C*_P_ on size for Ag, Al, and Cu nanoparticles of different shapes at 300 K, including spheres, tetrahedra, octahedra, icosahedra, cylindrical nanowires, and nanofilms. The calculated results are compared with previous theoretical models.^10,37,47–50,57–59^ It can be seen that, for all three materials, *C*_P_ increases rapidly as particle size decreases to a few nanometers and gradually approaches bulk material values as particle size increases. This nonlinear trend is particularly evident in the smaller size range of about 10 nm, where surface effects become strongly dominant in the nanosystem's thermal vibration properties. For Ag and Cu, the heat capacity increases sharply as the size decreases from about 20 nm to below 5 nm. Meanwhile, Al exhibited a significantly larger increase compared to Ag and Cu at the same size, reflecting the lower atomic bond energy and greater lattice vibrational softness of Al. Another important characteristic is the pronounced influence of geometric shape on isobaric heat capacity. The results show that tetrahedral and octahedral structures have the largest *C*_P_ values, followed by spherical and regular icosahedral particles, whereas nanowires and, especially, nanofilms have the smallest *C*_P_ values. This difference becomes more pronounced in the sub-5 nm size range. Physically, the increase in heat capacity at small sizes stems from a decrease in the average atomic coordination number and a weakening of the effective bond energy as the proportion of surface atoms increases. Surface atoms have unsaturated bonds, so they vibrate more strongly than atoms in the crystal core, increasing the system's ability to absorb thermal energy. Simultaneously, the decrease in the Debye temperature at the nanoscale also contributes to the theoretical increase in heat capacity predicted by Debye theory. However, As observed in Fig. 5, low-dimensional structures, such as nanowires and nanofilms, have significantly lower *C*_P_ values than three-dimensional nanostructures. This suggests that heat capacity depends not only on the amplitude of atomic vibrations but also strongly on the phonon density and the effective number of vibration modes of the system. In low-dimensional structures, phonon confinement in one or two spatial dimensions reduces the number of vibration modes that effectively contribute to heat capacity, thereby lowering the *C*_P_ value. Conversely, polyhedral structures such as tetrahedra and octahedra have higher atomic densities at edges and vertices, increasing local vibrations and the thermal energy absorption capacity of the nanosystem.

**Fig. 5:**
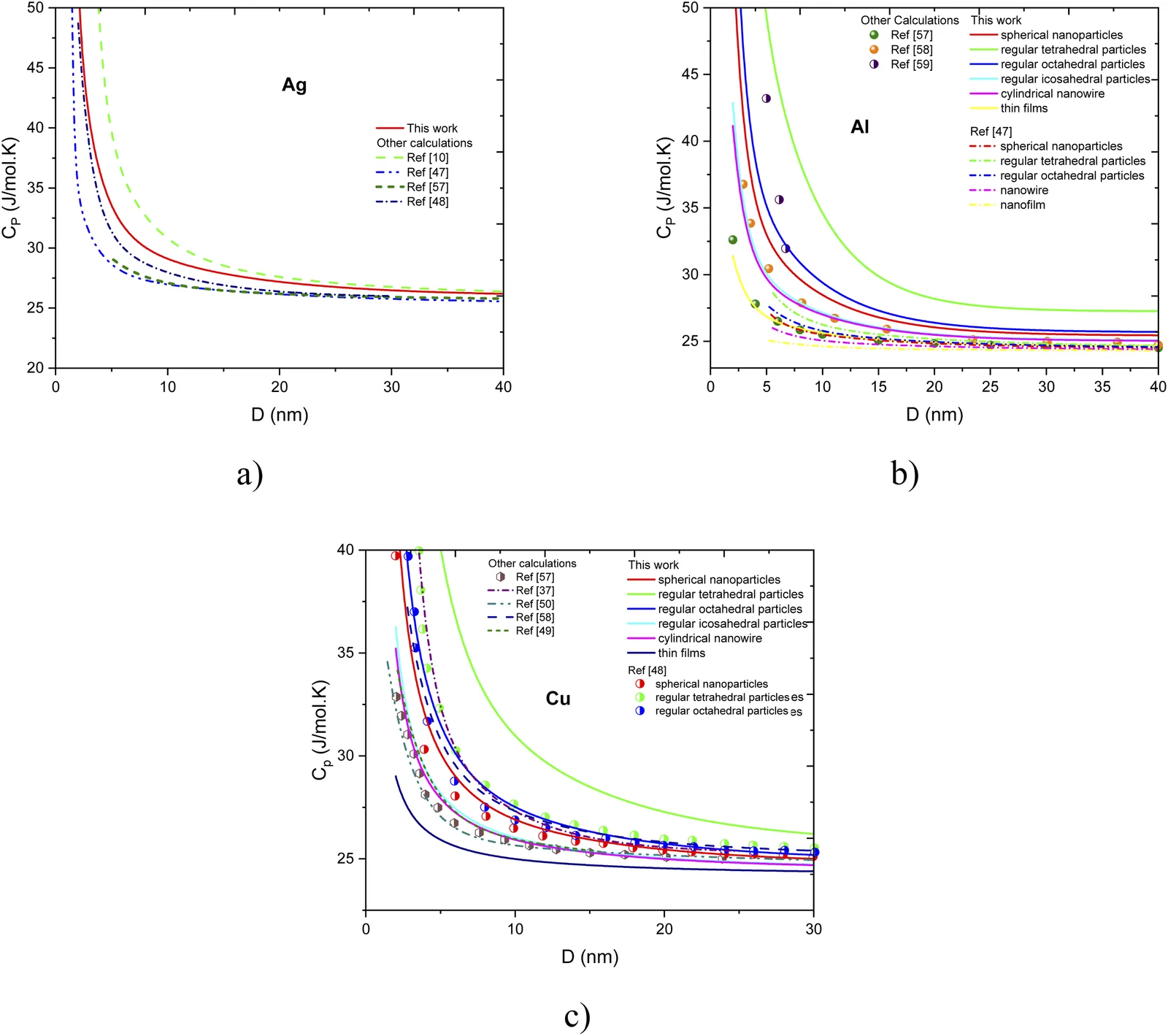
Isobaric heat capacity of Ag, Al, Cu nanoparticles depending on shape and size at temperature *T* = 300 K, respectively, is shown in figures (a–c) compared with theoretical calculations.^10,37,47–50,57–59^

Comparison with previous theoretical results shows that the current theoretical model fits well in both the trend of change and the quantitative value of isobaric heat capacity. The results show high similarity with the models of Zhu *et al.*,^37^ Singh *et al.*,^57^ and Khalaf *et al.*^58^ in the medium- and large-size regions. In particular, the strong upward trend of *C*_P_ in the small-size region observed in this study is consistent with the bonding-energy-based models of Singh^48^ and Ansari.^10^ However, certain differences still exist between the models in the size region below about 3 nm, especially for Al. The main reason stems from differences in the way surface bonding energy, average atomic coordination number, and the influence of geometric shape in each theoretical model. Some previous models used the isotropic oscillation assumption and therefore did not fully describe the influence of phonon anisotropy in low-dimensional structures such as nanowires and nanofilms. Furthermore, molecular dynamics simulations by Fedorov *et al.*^59^ show that surface atomic restructuring and local vibrations at the boundary region can significantly affect the heat capacity of very small nanoparticles, while analytical thermodynamic models often do not fully account for these effects. Therefore, further experimental results are needed to verify these theoretical models.

Fig. 6 and 7 present the dependence of *C*_P_ on temperature for Cu and Al nanoparticles. Fig. 6 investigates the influence of geometric shape on the *C*_P_ of Cu nanoparticles at 8 nm size, including spherical, tetrahedral, octahedral, icosahedral, nanowire, and nanofilm nanoparticles. The results are compared with the theoretical model of Zhu *et al.*^37^ and the experimental data of Rupp and Birringer.^25^Fig. 7 presents the dependence of *C*_P_ on temperature for spherical Al nanoparticles of different sizes from 5 nm to 30 nm, and compares them with experimental values of bulk materials.^51^ It can be seen that for both Cu and Al, the isobaric heat capacity increases with temperature in the investigated range. However, this increase is nonlinear and becomes more pronounced at higher temperatures. For Cu in Fig. 6, tetrahedral and octahedral structures exhibit the largest *C*_P_ values, followed by spherical and regular icosahedral particles, while nanowires and especially nanofilms have significantly lower values. The difference between the shapes increases progressively with increasing temperature from approximately 100 K to 400 K, suggesting a combined effect of surface effects and anharmonic thermal vibrations. In Fig. 7, for spherical Al, *C*_P_ increases with decreasing size. For example, at approximately 600 K, particles with a size of 5 nm give significantly larger *C*_P_ values than bulk materials, while particles of 15–30 nm give intermediate values and gradually converge towards the bulk limit as size increases. Physically, the increase in *C*_P_ with temperature stems from the increasing activation of lattice vibration modes as thermal energy increases. At low temperatures, only a fraction of the low-energy phonon modes contribute to the heat capacity, while at high temperatures, higher-energy vibration modes are gradually activated, increasing the system's thermal energy absorption. For small nanoparticles, a high surface-to-volume ratio reduces the average atomic coordination number and weakens the effective bonding energy. This leads to lattice vibration softening and increases the contribution of thermal vibrations to the heat capacity. Furthermore, the strong dependence on shape underscores the importance of phonon anisotropy and surface structure. Polyhedral structures such as tetrahedra and octahedra have higher atomic densities at edges and vertices, increasing local vibrations and enhancing thermal energy absorption. Conversely, in low-dimensional structures such as nanowires and nanofilms, the spatial phonon confinement effect reduces the number of vibration modes that effectively contribute to the heat capacity, resulting in lower *C*_P_ values. Comparison with previous results shows that the current model fits well with the trend of variation of both theoretical calculations^37^ and experimental data.^25,52^ For Cu, the calculated values fit well with the model of Zhu *et al.*,^37^ especially in the medium- and high-temperature regions. At the same time, the trend of increasing heat capacity of nanoparticles compared to bulk materials also fits with the experimental results of Rupp and Birringer.^25^ For Al, the calculated curves for bulk materials agree with the experimental data,^52^ whereas small nanoparticles exhibit greater deviations due to the strong influence of surface effects. The above results have important practical implications for the design of metal nanomaterials for thermal and energy applications. The strong dependence of *C*_P_ on size, shape, and temperature suggests that the heat-storage and heat-exchange capabilities of nanosystems can be tuned through morphological control. Structures with high heat capacity, such as tetrahedral or octahedral particles, are suitable for heat storage, thermocatalysis, and energy absorption applications, while low-dimensional structures, such as nanowires and nanofilms, have potential for nanoelectronic devices that require rapid thermal response and high thermal stability.

**Fig. 6:**
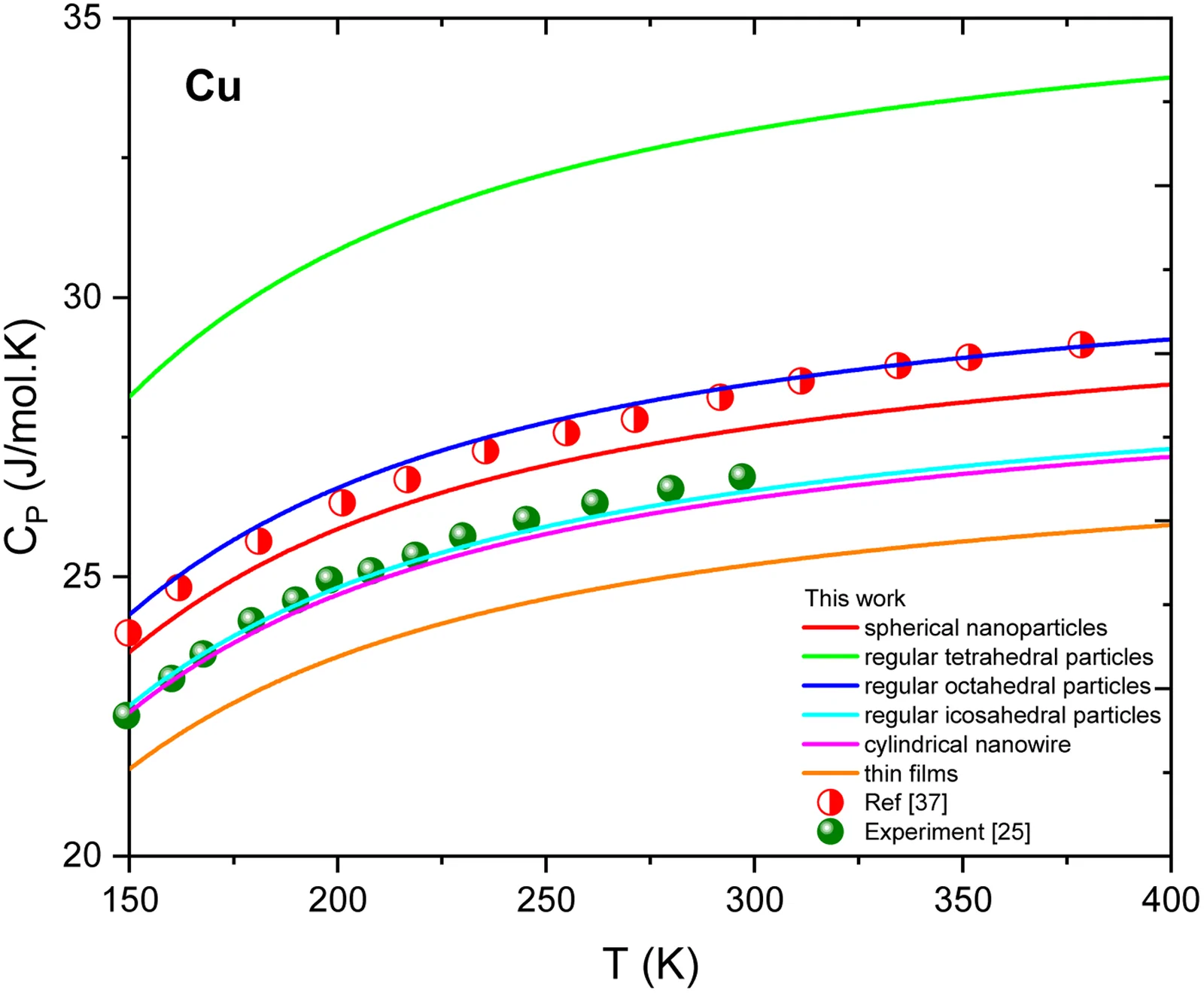
*C*_P_ of Cu nanoparticles depending on temperature and different shapes at 8 nm size compared with theoretical calculations^37^ and experimental.^25^

**Fig. 7:**
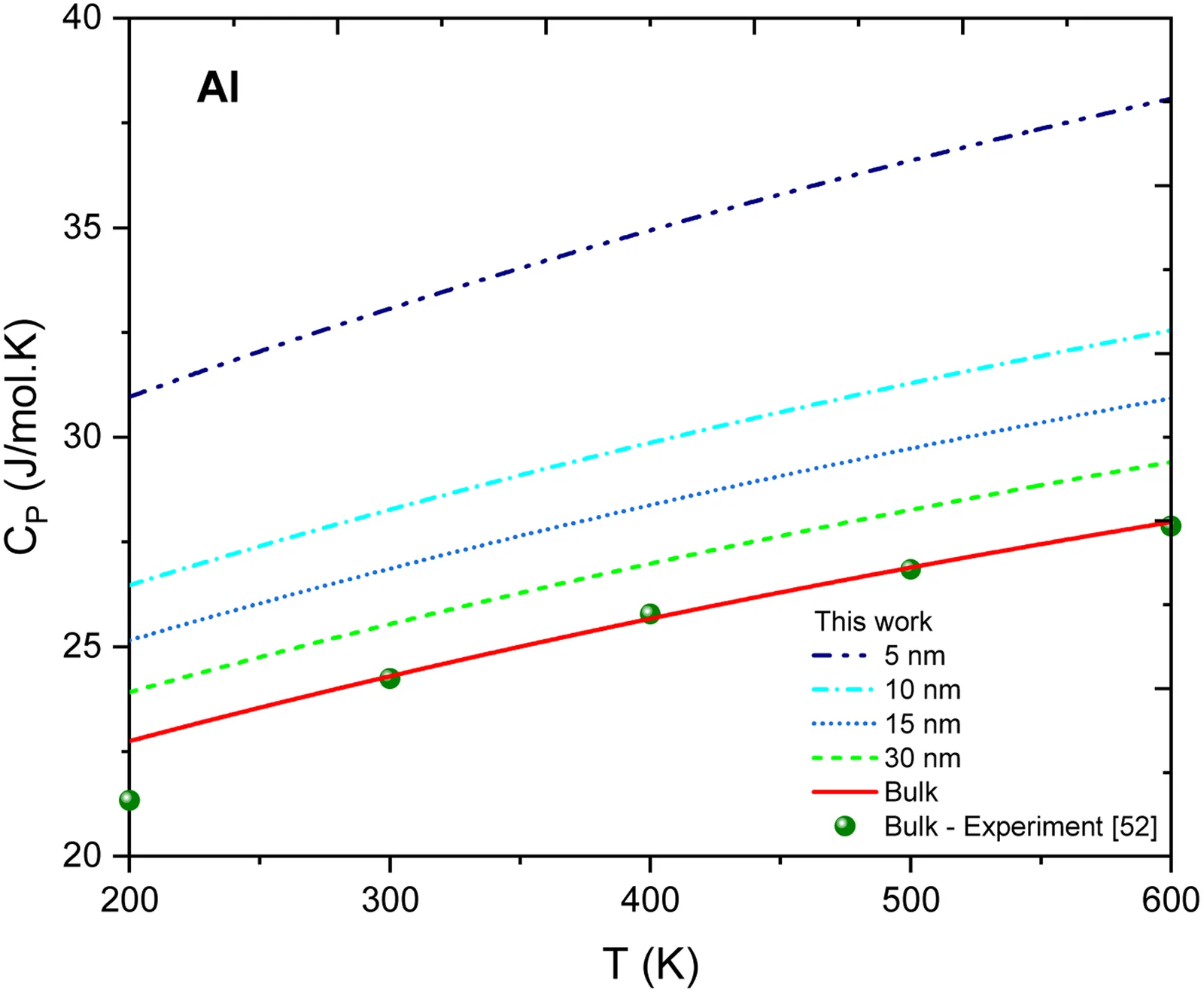
*C*_P_ of spherical Al nanoparticles depending on size and temperature compared with experimental.^52^

More broadly, previous atomistic studies have shown that the finite-temperature behavior of Ag and Cu clusters and Ag–Cu nanoalloys may involve competition between different structural motifs, structural transformations, chemical ordering, and thermally induced rearrangements.^60–63^ These studies provide complementary atomistic insight into nanoscale thermodynamics and structural evolution, whereas the present analytical treatment focuses on the systematic dependence of MSD, TEC, isobaric heat capacity, and Debye temperature on size, morphology, and temperature for prescribed nanostructures.

## 4 Conclusion

In this work, we formulated a theoretical framework combining the SMM with a bonding energy model to study the thermodynamic properties of Ag, Al, and Cu nanoparticles. We have derived analytical expressions for quantities such as MSD, TEC, isobaric heat capacity, and Debye temperature as functions of shape, size, and temperature. This formalism was systematically applied to nanoparticles ranging from 2 nm to 40 nm, 100 K ≤ *T* ≤ 1000 K across diverse morphologies, including spheres, regular tetrahedra, octahedra, icosahedra, nanowires, and thin films.

The results show that the thermodynamic properties of nanoparticles strongly depend on size, shape, and temperature. Specifically, as the particle size decreases, the MSD, TEC, and isobaric heat capacity exhibit a pronounced increase, whereas the Debye temperature correspondingly decreases. As particle size increases, the values of thermodynamic properties gradually approach those of bulk materials. At high temperatures, anharmonic effects significantly influence the thermodynamic properties of nanoparticles, especially MSD and TEC. This indicates that surface and anharmonic effects in lattice vibrations are indispensable for investigating the thermodynamic properties of nanoparticles smaller than 5 nm. Morphologically, the thermodynamic properties exhibit the strongest size-dependence in regular tetrahedral nanoparticles due to their high surface-to-volume ratio, whereas thin films demonstrate the weakest dependence. The robust agreement of our SMM calculations with both MD simulations and experimental data confirms the validity and reliability of our theoretical approach.

From an application-oriented perspective, the present results provide theoretical guidance for tailoring the thermal behavior of metallic nanostructures through control of size and morphology. The predicted variations in isobaric heat capacity may be useful for identifying structures with different heat-storage characteristics, while the size- and temperature-dependent TEC and MSD provide information relevant to thermal expansion and atomic thermal motion. These quantities alone do not determine the performance of a specific device, which also depends on thermal transport, interfaces, mechanical stability, and environmental conditions. Therefore, the present framework is best regarded as a low-cost theoretical screening tool for selecting candidate nanoparticle sizes and morphologies prior to detailed atomistic simulations and experimental evaluation.

## Conflicts of interest

There are no conflicts to declare.

## Data Availability

The authors confirm that the data supporting the findings of this study are available within the article. Raw data that support the findings of this study are available from the corresponding author, upon reasonable request.
